# Significance of Oligomeric and Fibrillar Species in Amyloidosis: Insights into Pathophysiology and Treatment

**DOI:** 10.3390/molecules26165091

**Published:** 2021-08-22

**Authors:** Haruki Koike, Yohei Iguchi, Kentaro Sahashi, Masahisa Katsuno

**Affiliations:** Department of Neurology, Graduate School of Medicine, Nagoya University, Nagoya 466-8550, Japan; iguyo@med.nagoya-u.ac.jp (Y.I.); sahashik@med.nagoya-u.ac.jp (K.S.); ka2no@med.nagoya-u.ac.jp (M.K.)

**Keywords:** AA amyloidosis, AL amyloidosis, Alzheimer’s disease, amyotrophic lateral sclerosis, ATTR amyloidosis, dementia, Parkinson’s disease, pathology, prion, transthyretin

## Abstract

Amyloidosis is a term referring to a group of various protein-misfolding diseases wherein normally soluble proteins form aggregates as insoluble amyloid fibrils. How, or whether, amyloid fibrils contribute to tissue damage in amyloidosis has been the topic of debate. In vitro studies have demonstrated the appearance of small globular oligomeric species during the incubation of amyloid beta peptide (Aβ). Nerve biopsy specimens from patients with systemic amyloidosis have suggested that globular structures similar to Aβ oligomers were generated from amorphous electron-dense materials and later developed into mature amyloid fibrils. Schwann cells adjacent to amyloid fibrils become atrophic and degenerative, suggesting that the direct tissue damage induced by amyloid fibrils plays an important role in systemic amyloidosis. In contrast, there is increasing evidence that oligomers, rather than amyloid fibrils, are responsible for cell death in neurodegenerative diseases, particularly Alzheimer’s disease. Disease-modifying therapies based on the pathophysiology of amyloidosis have now become available. Aducanumab, a human monoclonal antibody against the aggregated form of Aβ, was recently approved for Alzheimer’s disease, and other monoclonal antibodies, including gantenerumab, solanezumab, and lecanemab, could also be up for approval. As many other agents for amyloidosis will be developed in the future, studies to develop sensitive clinical scales for identifying improvement and markers that can act as surrogates for clinical scales should be conducted.

## 1. Introduction

Amyloidosis is a term referring to a group of toxic gain-of-function protein-misfolding diseases wherein normally soluble proteins aggregate in extracellular spaces as insoluble amyloid fibrils with a beta (β)-sheet structure [1,2]. More than 30 causative amyloidogenic proteins have been reported, and some of them, such as the amyloid β precursor protein (APP) in Alzheimer’s disease, prion protein in prion diseases, immunoglobulin light chain in AL amyloidosis, transthyretin (TTR) in ATTR amyloidosis, and serum amyloid A in AA amyloidosis, cause fatal outcomes [1,3,4,5,6,7,8]. The deposition of amyloid is localized to the central nervous system in Alzheimer’s disease and most prion diseases [1,3,4], whereas systemic deposition occurs in AL, ATTR, and AA amyloidoses [5,7,8,9,10]. How, or whether, amyloid fibrils contribute to these diseases is a topic of debate. The extracellular deposits, composed of amyloid fibrils (i.e., amyloid deposits), were initially regarded as the cause of organ dysfunction resulting from amyloidosis [11,12]. For example, the restriction of ventricular wall mobility due to massive amyloid deposition in the spaces between cardiomyocytes results in heart failure [9,13]. The direct damage of neighboring tissues by amyloid fibrils has also been suggested [11,12,14,15,16,17,18]. In contrast, more recent studies have focused on non-fibrillar precursors of amyloidogenic proteins as the cause of tissue degeneration [19,20,21]. In particular, protein oligomers generated during the process of amyloid fibril formation or released from amyloid fibril aggregates are now considered as causes of cellular dysfunction and degeneration [22,23,24,25]. In support of this view, the severity of cognitive decline in patients with Alzheimer’s disease does not correlate with amyloid plaque formation, suggesting that pre-amyloid aggregates are the cause of disease [26,27]. From this standpoint, clarifying the significance of amyloidogenic protein oligomers is important to understanding the pathophysiology and establishing therapeutic strategies for amyloidosis. 

In this review, we describe the pathophysiological aspects of amyloidosis, focusing on the prefibrillar states of amyloidogenic proteins and their evolution to amyloid fibrils. 

## 2. Initiation of Protein Aggregation 

The misfolding of proteins is an important step in the process of amyloid fibril formation [28]. In ATTR amyloidosis, TTR, which is mainly synthesized in the liver, forms amyloid fibrils due to the dissociation of natively folded tetramers into misfolded monomers [29,30]. In addition, proteolytic cleavage also promotes the misfolding and aggregation of TTR [31,32]. In Alzheimer’s disease, the proteolytic cleavage of APP by secretases results in the production of toxic amyloid β peptide (Aβ), which is prone to aggregation [33]. Furthermore, increased production, decreased clearance, oxidative modification, and phosphorylation of causative proteins are factors that may trigger the process of aggregation [2]. These factors are considered to play an important role in the initiation of protein aggregation in most acquired amyloidoses.

The formation of amyloid fibrils is a dynamic process, with monomers and oligomers being rapidly exchanged for each other depending on various factors that include pH, temperature, and co-solvents [34]. According to studies of serial biopsy specimens obtained from AL, ATTR, and AA amyloidosis patients, even mature amyloid fibril masses disappear when successful disease-modifying therapies are provided [35,36,37]. Electron microscope studies have demonstrated the appearance of dotty or globular structures 4 to 5 nm in diameter and the subsequent formation of short protofibrils 30 to 100 nm in length during an incubation of Aβ in vitro [38]. 

**Figure 1:**
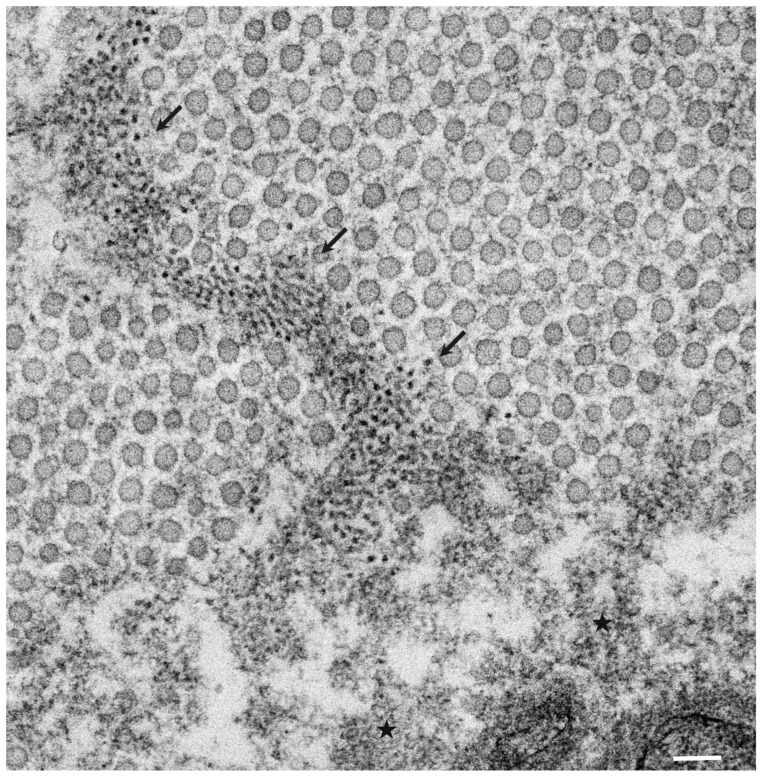
Amorphous electron-dense materials and clusters of globular structures in the endoneurium. A cross section of the sural nerve biopsy specimen from a patient with hereditary transthyretin (ATTRv) amyloidosis. Numerous small globular structures with a diameter of several nanometers seem to be generated from amorphous extracellular electron-dense materials supposed to contain amyloidogenic transthyretin. A cluster of globular structures and amorphous electron-dense materials are indicated by arrowheads and asterisks, respectively. The circular structures, with diameters of around 50 nm, are collagen fibers. Uranyl acetate and lead citrate stain. Scale bar = 0.1 μm.

The pathological studies of ATTR amyloidosis have also suggested a similar process of amyloid fibril formation via intermediates [7,17]. Observations of nerve biopsy specimens obtained from patients with hereditary ATTR (ATTRv; v for variant) amyloidosis using electron microscopy suggest that globular structures of similar diameter to Aβ intermediates were generated from amorphous electron-dense materials [7,17]. According to these studies, the deposition of amorphous electron-dense materials was observed in extracellular spaces of the endoneurium, particularly around the microvessels and subperineurial space [17]. These amorphous materials contain non-fibrillar TTR intermediates because they are stained with anti-TTR antibodies but not with Congo red [9]. Clusters of globular structures were also often observed among these extracellular amorphous materials (Figure 1) [17]. The deposition of TTR-positive but Congo red-negative non-fibrillar deposits was observed even in asymptomatic carriers with no amyloid deposits [39]. Similar non-fibrillar TTR deposits have also been reported in transgenic mouse and rat models of ATTRv amyloidosis [40,41]. As the disruption of the blood–nerve barrier of endoneurial microvessels was reported, the TTR in the endoneurium was believed to be derived from the bloodstream [16]. In vitro studies of TTR aggregation suggested that such globular structures are oligomeric intermediates that have cytotoxic effects [42]. 

## 3. Formation of Amyloid Fibrils from Intermediates

**Figure 2:**
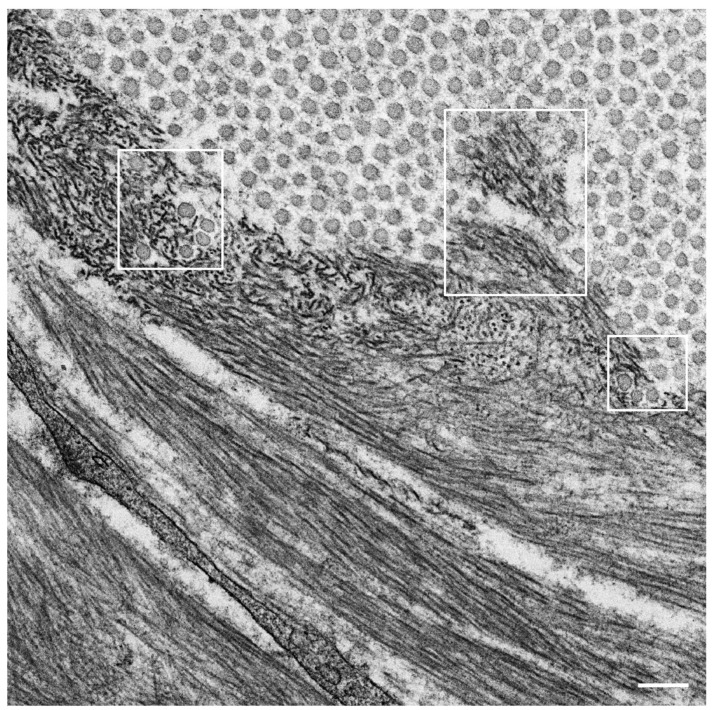
Amyloid fibrils in the endoneurium. A cross section of the sural nerve biopsy specimen from a patient with hereditary transthyretin (ATTRv) amyloidosis. The globular structures shown in Figure 1 seemed to develop into mature amyloid fibrils because elongated fibrillar structures are frequently found in the vicinity of such globular structures. The circular structures with diameters of around 50 nm are collagen fibers. Collagen fibers surrounded by squares seem to get involved in the aggregates of amyloid fibrils. Uranyl acetate and lead citrate stain. Scale bar = 0.1 μm.

**Figure 3:**
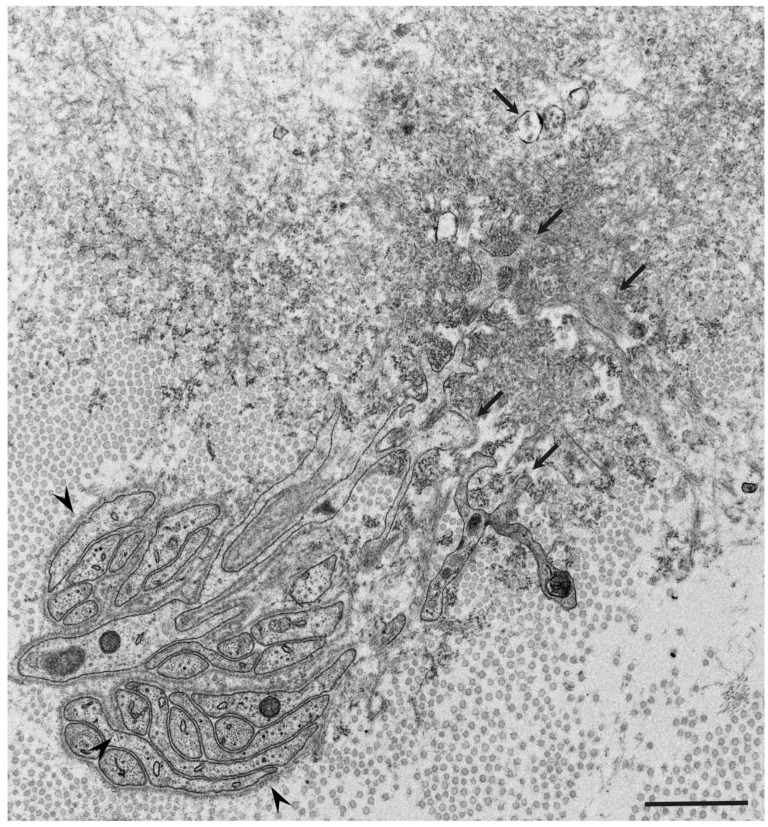
Atrophy and degeneration of Schwann cells apposed by amyloid fibrils. A cross section of a sural nerve biopsy specimen from a patient with hereditary transthyretin (ATTRv) amyloidosis. Schwann cells, particularly small ones associated with small-diameter nerve fibers, become atrophic when they are adjacent to amyloid fibril aggregates. Atrophy of Schwan cells is conspicuous in the upper right, where a mass of amyloid fibrils aggregation is present. The basement and cytoplasmic membranes apposed to amyloid fibrils become indistinct (arrows), while those in the lower left part, where amyloid fibrils are not present, are preserved (arrowheads). Uranyl acetate and lead citrate stain. Scale bar = 2 μm.

Observations of nerve biopsy specimens from patients with ATTRv amyloidosis using electron microscopy demonstrated a putative chronological sequence of the process of amyloid fibril formation and tissue damage [16,17]. The globular structures found in the extracellular electron-dense materials, which were described earlier, seemed to develop into mature amyloid fibrils because elongated fibrillar structures were frequently found in the vicinity of these globular structures (Figure 2) [17]. During the process of amyloid fibril maturation, amyloid fibrils affect the surrounding structures. For example, collagen fibers seem to get involved in the aggregates of amyloid fibrils, and the basement and cytoplasmic membranes of cells in the endoneurium, including Schwann cells, vascular endothelial cells, and pericytes, apposed by amyloid fibrils also become obscure and appear as if they fuse with amyloid fibrils (Figure 3) [21,43]. Additionally, Schwann cells, particularly small ones associated with small-diameter nerve fibers, become atrophic when they are adjacent to amyloid fibril masses [16,17]. Finally, the contours of these cells sometimes completely disappear, and only the cytoplasmic organelles of these cells remain among the aggregates of amyloid fibrils [17]. 

Similar atrophy of Schwann cells apposed by amyloid fibrils has also been reported in nerve biopsy specimens from patients with AL amyloidosis, which is another major type of systemic amyloidosis [10,14,15,18,44]. The atrophy and degeneration of vascular endothelial cells, pericytes, and neurites have also been found in brain biopsy specimens from patients with Alzheimer’s disease [45]. These findings support the concept that direct tissue damage induced by amyloid fibrils plays an important role in amyloidosis. In fact, the amount of amyloid deposits seems to be correlated with the extent of neurodegeneration in certain types of ATTRv amyloidosis, particularly the early-onset form of ATTRv amyloidosis [9,46]. However, severe nerve fiber loss was observed in the late-onset form of ATTRv amyloidosis despite a small amount of amyloid deposits, suggesting the presence of other factors for tissue damage [9]. From this standpoint, researchers are now paying attention to the toxicity of non-fibrillar oligomers in tissue damage associated with amyloidosis. 

## 4. The Role of Non-Fibrillar Oligomers in Tissue Damage

There is increasing evidence that non-fibrillar oligomers or protofibrils, rather than large fibrils, are responsible for cell death in common neurodegenerative diseases of the central nervous system, including Alzheimer’s disease, Parkinson’s disease, amyotrophic lateral sclerosis, Huntington’s disease, and spinocerebellar ataxia [20,23,47]. Some researchers have even hypothesized that the formation of mature amyloid fibrils is a protective process in these diseases [20]. For example, the soluble intermediates of Aβ in the brain correlated with the marker of disease severity in patients with Alzheimer’s disease; this was not true with insoluble Aβ [48]. Supporting these findings, human Aβ oligomers, but not amyloid fibrils, were found to have a synaptotoxic effect in vivo [19]. In patients with dementia resulting from APP E693Delta mutation, which confers the property of enhanced oligomerization but no fibrillization, a very low amyloid signal was observed on positron emission tomography [26]. Additionally, transgenic mice expressing the same APP mutation showed memory impairment and pathological findings similar to patients with Alzheimer’s disease despite the absence of amyloid plaques [27]. 

The toxicity of oligomers has also been suggested in systemic amyloidosis, including AL, ATTR, and AA amyloidoses [21,25,42,49]. However, direct stress by amyloid fibrils also seems to participate in the mechanisms of tissue damage in these amyloidoses. As described earlier, the atrophy and degeneration of tissues seem to occur in the peripheral nervous system along with the formation of amyloid fibrils [17]. Although mechanical stress generated during the maturation of amyloid fibrils may affect neighboring tissues [10,17], biochemical stress may also participate in the mechanisms of tissue damage. As the turnover of amyloid fibril components occurs steadily [23,36], soluble oligomers may be constantly present in the vicinity of amyloid fibrils. 

There are numerous reports on the mechanisms of tissue damage by non-fibrillar oligomers. Oligomers may directly affect cytoplasmic membranes by increasing membrane conductance and calcium influx, triggering the atrophy and degeneration of neighboring cells [22,24]. Normal cellular prion protein expressed on cell surface mediates the toxic effects as a receptor of Aβ oligomers, suggesting that it plays an important role in Aβ-oligomers-induced neurodegeneration [50,51]. Aβ oligomers also act on receptors crucial for neurotransmission, such as glutamate receptors and nicotinic acetylcholine receptors [52,53,54]. Furthermore, accumulating evidence suggests that intracellular oligomers of misfolded proteins are also toxic to cells via interaction with mitochondria and nucleus in various neurodegenerative diseases [55,56,57,58]. 

## 5. Therapeutic Insights

With the progress in understanding the mechanisms of amyloid fibril formation and tissue damage, several disease-modifying therapies based on the pathophysiology of amyloidosis have become available. These include agents to reduce or prevent the production of causative proteins such as chemotherapeutic agents against plasma cell dyscrasia for AL amyloidosis, short interfering RNA and antisense oligonucleotides to knockdown TTR for ATTR amyloidosis, and monoclonal antibodies against proinflammatory cytokines for AA amyloidosis [7,10,59,60]. Small molecules that stabilize TTR tetramers have also become available for ATTR amyloidosis [7,60]. These disease-modifying therapies significantly improved the prognosis of AL, ATTR, and AA amyloidoses. In particular, clinical trials of gene silencing agents such as patisiran (a short interfering RNA) and inotersen (an antisense oligonucleotide) for patients with ATTRv amyloidosis demonstrated excellent efficacy in terms of clinical scores and quality of life [61,62,63,64]. The safety profile of patisiran was acceptable [62], whereas glomerulonephritis and thrombocytopenia were reported as severe adverse events of inotersen [63], necessitating close monitoring of renal function and platelet count. To improve the safety profile of inotersen, a ligand-conjugated antisense oligonucleotide designed to facilitate receptor-mediated uptake by hepatocytes was designed [65,66].

In June 2021, aducanumab, a human monoclonal antibody against the aggregated form of Aβ, was approved by the US Food and Drug Administration for use in treating patients with Alzheimer’s disease [67]. Following intravenous infusion, this antibody can cross the blood–brain barrier and selectively bind to Aβ aggregates [68]. From the discussion regarding the toxicity of oligomers in Alzheimer’s disease, aducanumab can remove not only insoluble amyloid fibrils but also soluble oligomers. However, two phase 3 trials conducted for aducanumab were halted because the interim analyses did not indicate remarkable success of these trials as determined from the cognitive functions [69]. The drug was approved using the accelerated approval pathway based on the reduction of the level of amyloid plaques in the brain, which was not the primary endpoint of these clinical trials. However, reduction in the cognitive decline from the viewpoint of the amyloid hypothesis might have been expected [67]. Monoclonal antibodies against the components of amyloid deposits are also under development for the treatment of various types of systemic amyloidosis [10]. For Alzheimer’s disease, other monoclonal antibodies, including gantenerumab [70], solanezumab [71], and lecanemab [72], could also be up for approval. 

## 6. Summary and Conclusions

Amyloidosis refers to a group of various protein-misfolding diseases wherein normally soluble proteins aggregate as insoluble amyloid fibrils [2]. How, or whether, amyloid fibrils contribute to tissue damage in amyloidosis has been a topic of debate. Direct stress to neighboring tissues imposed by amyloid fibrils themselves has classically been suggested [15,16,17,18], whereas recent studies have focused on the non-fibrillar precursors of amyloidogenic proteins, particularly soluble oligomers, as the cause of tissue damage [24,25]. 

Electron microscope studies have demonstrated the appearance of globular structures 4 to 5 nm in diameter and the subsequent formation of short protofibrils 30 to 100 nm in length during the incubation of Aβ in vitro [38]. Observations of nerve biopsy specimens from patients with ATTRv amyloidosis have suggested that globular structures of similar diameter to Aβ intermediates were generated from amorphous electron-dense materials containing TTR [9,17]. In vitro studies of TTR aggregation have suggested that such globular structures are oligomeric intermediates possessing cytotoxic effects [42]. Nonetheless, direct stress by amyloid fibrils themselves also seems to participate in the mechanisms of tissue damage in amyloidosis, particularly systemic amyloidosis [16,17]. However, soluble oligomers generated as a result of the turnover of amyloid fibril components may be constantly present in the vicinity of amyloid fibrils [36], triggering the tissue damage [22,24,25]. 

Disease-modifying therapies based on the pathophysiology of amyloidosis, which include chemotherapeutic agents against plasma cell dyscrasia for AL amyloidosis, gene silencing agents to knockdown TTR for ATTR amyloidosis, and monoclonal antibodies against proinflammatory cytokines for AA amyloidosis, have now become available [7,10,59,60]. Recently, a human monoclonal antibody against the aggregated form of Aβ, aducanumab, was approved by the US Food and Drug Administration for Alzheimer’s disease [67]. Although phase 3 trials for aducanumab were halted, the drug was approved using the accelerated approval pathway based on the reduction of the level of amyloid plaques [67]. As many other agents based on the pathophysiology of amyloidosis will be developed in the future, studies to develop sensitive clinical scales for identifying improvement and markers that can act as surrogates for clinical scales should be conducted.

## Data Availability

Not applicable.

## References

[1] Benson M.D., Buxbaum J.N., Eisenberg D.S., Merlini G., Saraiva M.J.M., Sekijima Y., Sipe J.D., Westermark P. (2020). Amyloid nomenclature 2020: Update and recommendations by the International Society of Amyloidosis (ISA) nomenclature committee. Amyloid.

[2] Ross C.A., Poirier M.A. (2004). Protein aggregation and neurodegenerative disease. Nat. Med..

[3] Scheltens P., Blennow K., Breteler M.M., de Strooper B., Frisoni G.B., Salloway S., Van der Flier W.M. (2016). Alzheimer’s disease. Lancet.

[4] Sigurdson C.J., Bartz J.C., Glatzel M. (2019). Cellular and Molecular Mechanisms of Prion Disease. Annu. Rev. Pathol..

[5] Perfetto F., Moggi-Pignone A., Livi R., Tempestini A., Bergesio F., Matucci-Cerinic M. (2010). Systemic amyloidosis: A challenge for the rheumatologist. Nat. Rev. Rheumatol..

[6] Koike H., Tanaka F., Hashimoto R., Tomita M., Kawagashira Y., Iijima M., Fujitake J., Kawanami T., Kato T., Yamamoto M. (2012). Natural history of transthyretin Val30Met familial amyloid polyneuropathy: Analysis of late-onset cases from non-endemic areas. J. Neurol. Neurosurg. Psychiatry.

[7] Koike H., Katsuno M. (2020). Transthyretin Amyloidosis: Update on the Clinical Spectrum, Pathogenesis, and Disease-Modifying Therapies. Neurol. Ther..

[8] Obici L., Adams D. (2020). Acquired and inherited amyloidosis: Knowledge driving patients’ care. J. Peripher. Nerv. Syst..

[9] Koike H., Misu K., Sugiura M., Iijima M., Mori K., Yamamoto M., Hattori N., Mukai E., Ando Y., Ikeda S. (2004). Pathology of early- vs late-onset TTR Met30 familial amyloid polyneuropathy. Neurology.

[10] Koike H., Katsuno M. (2021). The Ultrastructure of Tissue Damage by Amyloid Fibrils. Molecules.

[11] Coimbra A., Andrade C. (1971). Familial amyloid polyneuropathy: An electron microscope study of the peripheral nerve in five cases. I. Interstitial changes. Brain.

[12] Thomas P.K., King R.H. (1974). Peripheral nerve changes in amyloid neuropathy. Brain.

[13] Koike H., Okumura T., Murohara T., Katsuno M. (2021). Multidisciplinary Approaches for Transthyretin Amyloidosis. Cardiol. Ther..

[14] Vital C., Vallat J.M., Deminiere C., Loubet A., Leboutet M.J. (1982). Peripheral nerve damage during multiple myeloma and Waldenstrom’s macroglobulinemia: An ultrastructural and immunopathologic study. Cancer.

[15] Sommer C., Schröder J.M. (1989). Amyloid neuropathy: Immunocytochemical localization of intra- and extracellular immunoglobulin light chains. Acta Neuropathol..

[16] Koike H., Ikeda S., Takahashi M., Kawagashira Y., Iijima M., Misumi Y., Ando Y., Ikeda S.-I., Katsuno M., Sobue G. (2016). Schwann cell and endothelial cell damage in transthyretin familial amyloid polyneuropathy. Neurology.

[17] Koike H., Nishi R., Ikeda S., Kawagashira Y., Iijima M., Sakurai T., Shimohata T., Katsuno M., Sobue G. (2018). The morphology of amyloid fibrils and their impact on tissue damage in hereditary transthyretin amyloidosis: An ultrastructural study. J. Neurol. Sci..

[18] Koike H., Mouri N., Fukami Y., Iijima M., Matsuo K., Yagi N., Saito A., Nakamura H., Takahashi K., Nakae Y. (2021). Two distinct mechanisms of neuropathy in immunoglobulin light chain (AL) amyloidosis. J. Neurol. Sci..

[19] Walsh D.M., Klyubin I., Fadeeva J.V., Cullen W.K., Anwyl R., Wolfe M.S., Rowan M.J., Selkoe D.J. (2002). Naturally secreted oligomers of amyloid beta protein potently inhibit hippocampal long-term potentiation in vivo. Nature.

[20] Caughey B., Lansbury P.T. (2003). Protofibrils, pores, fibrils, and neurodegeneration: Separating the responsible protein aggregates from the innocent bystanders. Annu. Rev. Neurosci..

[21] Koike H., Katsuno M. (2019). Ultrastructure in Transthyretin Amyloidosis: From Pathophysiology to Therapeutic Insights. Biomedicines.

[22] Kayed R., Sokolov Y., Edmonds B., McIntire T.M., Milton S.C., Hall J.E., Glabe C.G. (2004). Permeabilization of lipid bilayers is a common conformation-dependent activity of soluble amyloid oligomers in protein misfolding diseases. J. Biol. Chem..

[23] Benilova I., Karran E., De Strooper B. (2012). The toxic Aβ oligomer and Alzheimer’s disease: An emperor in need of clothes. Nat. Neurosci..

[24] Evangelisti E., Cascella R., Becatti M., Marrazza G., Dobson C.M., Chiti F., Stefani M., Cecchi C. (2016). Binding affinity of amyloid oligomers to cellular membranes is a generic indicator of cellular dysfunction in protein misfolding diseases. Sci. Rep..

[25] Jayaraman S., Gantz D.L., Haupt C., Gursky O. (2017). Serum amyloid A forms stable oligomers that disrupt vesicles at lysosomal pH and contribute to the pathogenesis of reactive amyloidosis. Proc. Natl. Acad. Sci. USA.

[26] Tomiyama T., Nagata T., Shimada H., Teraoka R., Fukushima A., Kanemitsu H., Takuma H., Kuwano R., Imagawa M., Ataka S. (2008). A new amyloid beta variant favoring oligomerization in Alzheimer’s-type dementia. Ann. Neurol..

[27] Tomiyama T., Matsuyama S., Iso H., Umeda T., Takuma H., Ohnishi K., Ishibashi K., Teraoka R., Sakama N., Yamashita T. (2010). A mouse model of amyloid beta oligomers: Their contribution to synaptic alteration, abnormal tau phosphorylation, glial activation, and neuronal loss in vivo. J. Neurosci..

[28] Eisele Y.S., Monteiro C., Fearns C., Encalada S.E., Wiseman R.L., Powers E.T., Kelly J.W. (2015). Targeting protein aggregation for the treatment of degenerative diseases. Nat. Rev. Drug Discov..

[29] Blake C.C., Geisow M.J., Swan I.D., Rerat C., Rerat B. (1974). Strjcture of human plasma prealbumin at 2-5 A resolution. A preliminary report on the polypeptide chain conformation, quaternary structure and thyroxine binding. J. Mol. Biol..

[30] Kelly J.W. (1997). Amyloid fibril formation and protein misassembly: A structural quest for insights into amyloid and prion diseases. Structure.

[31] Mangione P.P., Verona G., Corazza A., Marcoux J., Canetti D., Giorgetti S., Raimondi S., Stoppini M., Esposito M., Relini A. (2018). Plasminogen activation triggers transthyretin amyloidogenesis in vitro. J. Biol. Chem..

[32] Dasari A.K.R., Arreola J., Michael B., Griffin R., Kelly J.W., Lim K.H. (2020). Disruption of the CD Loop by Enzymatic Cleavage Promotes the Formation of Toxic Transthyretin Oligomers through a Common Transthyretin Misfolding Pathway. Biochemistry.

[33] Selkoe D.J., Hardy J. (2016). The amyloid hypothesis of Alzheimer’s disease at 25 years. EMBO Mol. Med..

[34] Buell A.K., Dobson C.M., Knowles T.P. (2014). The physical chemistry of the amyloid phenomenon: Thermodynamics and kinetics of filamentous protein aggregation. Essays Biochem..

[35] Okuda Y., Takasugi K. (2006). Successful use of a humanized anti-interleukin-6 receptor antibody, tocilizumab, to treat amyloid A amyloidosis complicating juvenile idiopathic arthritis. Arthritis Rheum..

[36] Tsuchiya-Suzuki A., Yazaki M., Sekijima Y., Kametani F., Ikeda S.-I. (2013). Steady turnover of amyloid fibril proteins in gastric mucosa after liver transplantation in familial amyloid polyneuropathy. Amyloid.

[37] Katoh N., Matsushima A., Kurozumi M., Matsuda M., Ikeda S.-I. (2014). Marked and Rapid Regression of Hepatic Amyloid Deposition in a Patient with Systemic Light Chain (AL) Amyloidosis after High-dose Melphalan Therapy with Stem Cell Transplantation. Intern. Med..

[38] Nybo M., Svehag S.E., Nielsen E.H. (1999). An ultrastructural study of amyloid intermediates in A beta1-42 fibrillogenesis. Scand. J. Immunol..

[39] Sousa M.M., Cardoso I., Fernandes R., Guimarães A., Saraiva M.J. (2001). Deposition of transthyretin in early stages of familial amyloidotic polyneuropathy: Evidence for toxicity of nonfibrillar aggregates. Am. J. Pathol..

[40] Sousa M.M., Fernandes R., Palha J.A., Taboada A., Vieira P., Saraiva M.J. (2002). Evidence for early cytotoxic aggregates in transgenic mice for human transthyretin Leu55Pro. Am. J. Pathol..

[41] Ueda M., Ando Y., Hakamata Y., Nakamura M., Yamashita T., Obayashi K., Himeno S., Inoue S., Sato Y., Kaneko T. (2006). A transgenic rat with the human ATTR V30M: A novel tool for analyses of ATTR metabolisms. Biochem. Biophys. Res. Commun..

[42] Dasari A.K.R., Hughes R.M., Wi S., Hung I., Gan Z., Kelly J.W., Lim K.H. (2019). Transthyretin Aggregation Pathway toward the Formation of Distinct Cytotoxic Oligomers. Sci. Rep..

[43] Koike H., Fukami Y., Nishi R., Kawagashira Y., Iijima M., Sobue G., Katsuno M. (2019). Clinicopathological spectrum and recent advances in the treatment of hereditary transthyretin amyloidosis. Neurol. Clin. Neurosci..

[44] Vallat J.-M., Vital A., Magy L., Martin-Négrier M.-L., Vital C. (2009). An Update on Nerve Biopsy. J. Neuropathol. Exp. Neurol..

[45] Wisniewski H.M., Wegiel J., Wang K.C., Lach B. (1992). Ultrastructural studies of the cells forming amyloid in the cortical vessel wall in Alzheimer’s disease. Acta Neuropathol..

[46] Sobue G., Nakao N., Murakami K., Yasuda T., Sahashi K., Mitsuma T., Sasaki H., Sakaki Y., Takahashi A. (1990). Type I familial amyloid polyneuropathy. A pathological study of the peripheral nervous system. Brain.

[47] Choi M.L., Gandhi S. (2018). Crucial role of protein oligomerization in the pathogenesis of Alzheimer’s and Parkinson’s diseases. FEBS J..

[48] McLean C.A., Cherny R.A., Fraser F.W., Fuller S.J., Smith M.J., Beyreuther K., Bush A.I., Masters C.L. (1999). Soluble pool of Abeta amyloid as a determinant of severity of neurodegeneration in Alzheimer’s disease. Ann. Neurol..

[49] Clos A.L., Lasagna-Reeves C.A., Castillo-Carranza D.L., Sengupta U., Jackson G.R., Kelly B., Beachkofsky T.M., Kayed R. (2011). Formation of immunoglobulin light chain amyloid oligomers in primary cutaneous nodular amyloidosis. Br. J. Dermatol..

[50] Laurén J., Gimbel D.A., Nygaard H.B., Gilbert J.W., Strittmatter S.M. (2009). Cellular prion protein mediates impairment of synaptic plasticity by amyloid-beta oligomers. Nature.

[51] Zhang Y., Zhao Y., Zhang L., Yu W., Wang Y., Chang W. (2019). Cellular Prion Protein as a Receptor of Toxic Amyloid-β42 Oligomers Is Important for Alzheimer’s Disease. Front. Cell. Neurosci..

[52] Decker H., Jürgensen S., Adrover M.F., Brito-Moreira J., Bomfim T.R., Klein W.L., Epstein A.L., De Felice F.G., Jerusalinsky D., Ferreira S.T. (2010). N-Methyl-d-aspartate receptors are required for synaptic targeting of Alzheimer’s toxic amyloid-β peptide oligomers. J. Neurochem..

[53] Sun J.L., Stokoe S.A., Roberts J.P., Sathler M.F., Nip K.A., Shou J., Ko K., Tsunoda S., Kim S. (2019). Co-activation of selective nicotinic acetylcholine receptors is required to reverse beta amyloid-induced Ca^2+^ hyperexcitation. Neurobiol. Aging.

[54] Li S., Selkoe D.J. (2020). A mechanistic hypothesis for the impairment of synaptic plasticity by soluble Aβ oligomers from Alzheimer’s brain. J. Neurochem..

[55] Caspersen C., Wang N., Yao J., Sosunov A., Chen X., Lustbader J.W., Xu H.W., Stern D., McKhann G., Yan S.D. (2005). Mitochondrial Abeta: A potential focal point for neuronal metabolic dysfunction in Alzheimer’s disease. FASEB J..

[56] Kitamura A., Kubota H. (2010). Amyloid oligomers: Dynamics and toxicity in the cytosol and nucleus. FEBS J..

[57] Cascella R., Chen S.W., Bigi A., Camino J.D., Xu C.K., Dobson C.M., Chiti F., Cremades N., Cecchi C. (2021). The release of toxic oligomers from α-synuclein fibrils induces dysfunction in neuronal cells. Nat. Commun..

[58] Gonzalez-Garcia M., Fusco G., De Simone A. (2021). Membrane Interactions and Toxicity by Misfolded Protein Oligomers. Front. Cell Dev. Biol..

[59] Wechalekar A.D., Gillmore J.D., Hawkins P.N. (2016). Systemic amyloidosis. Lancet.

[60] Adams D., Koike H., Slama M., Coelho T. (2019). Hereditary transthyretin amyloidosis: A model of medical progress for a fatal disease. Nat. Rev. Neurol..

[61] Adams D., Gonzalez-Duarte A., O’Riordan W.D., Yang C.C., Ueda M., Kristen A.V., Tournev I., Schmidt H.H., Coelho T., Berk J.L. (2018). Patisiran, an RNAi Therapeutic, for Hereditary Transthyretin Amyloidosis. N. Engl. J. Med..

[62] Adams D., Polydefkis M., González-Duarte A., Wixner J., Kristen A.V., Schmidt H.H., Berk J.L., Losada López I.A., Dispenzieri A., Quan D. (2021). Long-term safety and efficacy of patisiran for hereditary transthyretin-mediated amyloidosis with polyneuropathy: 12-month results of an open-label extension study. Lancet Neurol..

[63] Benson M.D., Waddington-Cruz M., Berk J.L., Polydefkis M., Dyck P.J., Wang A.K., Planté-Bordeneuve V., Barroso F.A., Merlini G., Obici L. (2018). Inotersen Treatment for Patients with Hereditary Transthyretin Amyloidosis. N. Engl. J. Med..

[64] Coelho T., Yarlas A., Waddington-Cruz M., White M.K., Kessler A.S., Lovley A., Pollock M., Guthrie S., Ackermann E.J., Hughes S.G. (2019). Inotersen preserves or improves quality of life in hereditary transthyretin amyloidosis. J. Neurol..

[65] Viney N.J., Guo S., Tai L., Baker B.F., Aghajan M., Jung S.W., Yu R.Z., Booten S., Murray H., Machemer T. (2021). Ligand conjugated antisense oligonucleotide for the treatment of transthyretin amyloidosis: Preclinical and phase 1 data. ESC Heart Fail..

[66] Coelho T., Ando Y., Benson M.D., Berk J.L., Waddington-Cruz M., Dyck P.J., Gillmore J.D., Khella S.L., Litchy W.J., Obici L. (2021). Design and Rationale of the Global Phase 3 NEURO-TTRansform Study of Antisense Oligonucleotide AKCEA-TTR-LRx (ION-682884-CS3) in Hereditary Transthyretin-Mediated Amyloid Polyneuropathy. Neurol. Ther..

[67] Mullard A. (2021). FDA approval for Biogen’s aducanumab sparks Alzheimer disease firestorm. Nat. Rev. Drug Discov..

[68] Sevigny J., Chiao P., Bussière T., Weinreb P.H., Williams L., Maier M., Dunstan R., Salloway S., Chen T., Ling Y. (2016). The antibody aducanumab reduces Aβ plaques in Alzheimer’s disease. Nature.

[69] Knopman D.S., Jones D.T., Greicius M.D. (2020). Failure to demonstrate efficacy of aducanumab: An analysis of the EMERGE and ENGAGE trials as reported by Biogen, December 2019. Alzheimer’s Dement..

[70] Ostrowitzki S., Lasser R.A., Dorflinger E., Scheltens P., Barkhof F., Nikolcheva T., Ashford E., Retout S., Hofmann C., Delmar P. (2017). A phase III randomized trial of gantenerumab in prodromal Alzheimer’s disease. Alzheimer’s Res. Ther..

[71] Honig L.S., Vellas B., Woodward M., Boada M., Bullock R., Borrie M., Hager K., Andreasen N., Scarpini E., Liu-Seifert H. (2018). Trial of Solanezumab for Mild Dementia Due to Alzheimer’s Disease. N. Engl. J. Med..

[72] Swanson C.J., Zhang Y., Dhadda S., Wang J., Kaplow J., Lai R.Y.K., Lannfelt L., Bradley H., Rabe M., Koyama A. (2021). A randomized, double-blind, phase 2b proof-of-concept clinical trial in early Alzheimer’s disease with lecanemab, an anti-Aβ protofibril antibody. Alzheimer’s Res. Ther..

